# Defining a screening tool for post-traumatic stress disorder in East Africa: a penalized regression approach

**DOI:** 10.3389/fpubh.2024.1383171

**Published:** 2024-06-13

**Authors:** Susan M. Meffert, Muthoni A. Mathai, Linnet Ongeri, Thomas C. Neylan, Daniel Mwai, Dickens Onyango, Dickens Akena, Grace Rota, Ammon Otieno, Raymond R. Obura, Josline Wangia, Elizabeth Opiyo, Peter Muchembre, Dennis Oluoch, Raphael Wambura, Anne Mbwayo, James G. Kahn, Craig R. Cohen, David E. Bukusi, Gregory A. Aarons, Rachel L. Burger, Chengshi Jin, Charles E. McCulloch, Simon Njuguna Kahonge

**Affiliations:** ^1^Department of Psychiatry and Behavioral Sciences, University of California, San Francisco, San Francisco, CA, United States; ^2^Department of Psychiatry, University of Nairobi, Nairobi, Kenya; ^3^Kenya Medical Research Institute, Nairobi, Kenya; ^4^Department of Health Economics, University of Nairobi, Nairobi, Kenya; ^5^Kisumu County Ministry of Health, Kisumu, Kenya; ^6^Department of Psychiatry, Makerere University, Kampala, Uganda; ^7^Global Programs for Research and Training, Nairobi, Kenya; ^8^University of Nairobi, Nairobi, Kenya; ^9^Institute for Health Policy Studies, University of California, San Francisco, San Francisco, CA, United States; ^10^Department of Epidemiology and Biostatistics, University of California, San Francisco, San Francisco, CA, United States; ^11^Department of Obstetrics, Gynecology and Reproductive Sciences, University of California, San Francisco, San Francisco, CA, United States; ^12^Department of Psychiatry, University of California, San Diego, La Jolla, CA, United States; ^13^Kenya Ministry of Health, Nairobi, Kenya

**Keywords:** posttraumatic stress disorder (PTSD), East Africa (Kenya), screening tools, primary care, low and middle income countries (LMIC), traumatic stress, sub Saharan Africa

## Abstract

**Background:**

Scalable PTSD screening strategies must be brief, accurate and capable of administration by a non-specialized workforce.

**Methods:**

We used PTSD as determined by the structured clinical interview as our gold standard and considered predictors sets of (a) Posttraumatic Stress Checklist-5 (PCL-5), (b) Primary Care PTSD Screen for the DSM-5 (PC-PTSD) and, (c) PCL-5 and PC-PTSD questions to identify the optimal items for PTSD screening for public sector settings in Kenya. A logistic regression model using LASSO was fit by minimizing the average squared error in the validation data. Area under the receiver operating characteristic curve (AUROC) measured discrimination performance.

**Results:**

Penalized regression analysis suggested a screening tool that sums the Likert scale values of two PCL-5 questions—intrusive thoughts of the stressful experience (#1) and insomnia (#21). This had an AUROC of 0.85 (using hold-out test data) for predicting PTSD as evaluated by the MINI, which outperformed the PC-PTSD. The AUROC was similar in subgroups defined by age, sex, and number of categories of trauma experienced (all AUROCs>0.83) except those with no trauma history- AUROC was 0.78.

**Conclusion:**

In some East African settings, a 2-item PTSD screening tool may outperform longer screeners and is easily scaled by a non-specialist workforce.

## Introduction

### Mental health and trauma disorder treatment gap in Sub-Saharan Africa

The Global Burden of Disease (GBD) studies, launched in 1996, were some of the first studies of health disability and illuminated the massive, worldwide health impact of mental disorders (1). Although mental disorder disability is driven overwhelmingly by common mental disorders such as depression, anxiety and trauma-related conditions (2, 3) which have well established treatments, access to treatment is so limited in low- and middle-income countries (LMICs) that an extraordinarily 75% of people with serious mental disorders never receive any treatment at all (“treatment gap”) (4). The situation in Sub-Saharan Africa (SSA) is particularly extreme, with the treatment gap reaching over 90% in some regions (5, 6). Epidemiologic models predict that the disability burden from mental disorders in SSA will increase by 130% in the next 40 years (7, 8).

Posttraumatic Stress Disorder (PTSD) in SSA is driven by the high incidence of traumatic stressors, including armed conflict, political violence, traumatic bereavement and domestic violence (9). Estimates of probable PTSD in the general population in SSA reach as high as 30% (9). Reduction of PTSD at a population level requires both scalable models of evidence-based treatment and practical screening tools. Sustainable strategies for improving public sector access to first line PTSD care delivered by locally available, non-specialist providers have progressed in recent years, including studies in SSA (10, 11). However, the ability to scale up care is hampered by the lack of pragmatic and scalable PTSD screening measures that have been validated in these settings (12). Screening tools are not expected to improve mental conditions. Rather, they serve to identify individuals in need of treatment—the first, crucial step toward recovery.

### Study goal

The goal of this study was to develop a practical screening instrument that can be used to identify adults with probable PTSD in East Africa—the first step toward closing the PTSD treatment gap. We leveraged data from our implementation research study in Kenya. Using a structured diagnostic interview as a gold standard, we test items from the Posttraumatic Stress Checklist-5 (PCL-5, 20 items) (13) and the Primary Care PTSD Screen for the DSM-5 (PC-PTSD, 5 items), a commonly used PTSD screen in High Income Countries (HICs) (14).

## Methods

### Screening for post-traumatic stress disorders in East Africa

We ran a large implementation science study of scalable strategies for delivering major depression and/or PTSD treatment in western Kenya (*n* = 2,162): the Sequential, Multiple Assessment Randomized Trial (SMART) for non-specialist treatment of common mental disorders in Kenya: Leveraging the Depression And Primary care Partnership for Effectiveness-implementation Research (DAPPER) (15). As part of SMART DAPPER activities, we sought to identify a practical PTSD screening instrument that could be used by existing clinical staff for regional hospitals seeking to initiate their own mental health treatment programs.

Cultural differences are well-known to affect the experience and expression of mental disorders, and trauma-disorders have some of the highest variability (16–18). SMART DAPPER uses three different measures of PTSD and assesses for convergent validity. All measures are translated to local languages of Dhluo and Kiswahili, using established methodology (19).

*Mini international neuropsychiatric interview (MINI 7.0.2)-PTSD module* (20)*:* The current version of the PTSD module queries PTSD symptoms per DSM-5 diagnostic criteria, over the past month. While we regard the MINI as our gold standard for assessment of PTSD, it is too lengthy to be used as a screening instrument at scale.

*Posttraumatic stress checklist*−*5 (PCL-5)* (13)*:* The PCL-5 is a self-report questionnaire to assess symptoms of PTSD based on DSM-5 criteria (13): The PCL-5 includes 20 questions that measure DSM-5 Criteria B-E over the past month, with each question measuring symptom severity on a Likert scale from 0 (not at all) to 4 (extremely), and total scale ranging from 0 to 80.

The *primary care-PTSD-5 screen (PC-PTSD-5)* is a short PTSD screen based on DSM-5 criteria (14). The PC-PTSD-5 includes 5 questions that measure DSM-5 Criteria B-E over the past month, with each question on a binary scale (1 = Yes or 0 = No). Items are summed with a range from 0 to 5.

*Trauma history questionnaire (THQ)* (21)*:* The THQ consists of 24 items and assesses lifetime exposure to potentially traumatic events in the following categories: crime, general disaster, physical/sexual assault, and other. Given the association between trauma exposure number and type with risk of PTSD (22, 23) we scored the THQ according to the totals, sub-types and number of different types of lifetime trauma: 0, 1, 2, 3 or more (Table 1).

**Table 1:** Descriptive statistics for individuals included in the training, validation, and hold-out test data.^*^

**Variable**	**Training data (*n =* 2,112)**	**Validation data (*n =* 1,766)**	**Hold-out test data (*n =* 1,754)**	**Total (*n =* 5,632)**
Age in Years [Mean ± SD *(n)*]	35.7 ± 11 (*n =* 2,112)	35.7 ± 10.9 (*n =* 1,766)	35.7 ± 10.8 (*n =* 1,754)	35.7 ± 10.9 (*n =* 5,632)
Age in Years [Median (min-max)]	34 (18–85)	34 (18–85)	34 (18–84)	34 (18–85)
**Gender**				
Male	201 (9.5%)	161 (9.1%)	166 (9.5%)	528 (9.4%)
Female	1,911 (90.5%)	1,605 (90.9%)	1,588 (90.5%)	5,104 (90.6%)
Total	2,112	1,766	1,754	5,632
**Education**				
None	35 (1.7%)	31 (1.8%)	26 (1.5%)	92 (1.6%)
Some primary/primary	1,092 (51.7%)	923 (52.3%)	913 (52.1%)	2,928 (52.0%)
Some secondary/secondary	785 (37.2%)	648 (36.7%)	655 (37.3%)	2,088 (37.1%)
Some college/Certificate/Diploma/Degree/ Post-graduate	200 (9.5%)	164 (9.3%)	160 (9.1%)	524 (9.3%)
Total	2,112	1,766	1,754	5,632
**Major depression**				
Negative	142 (6.7%)	128 (7.2%)	115 (6.6%)	385 (6.8%)
Positive	1,970 (93.3%)	1,638 (92.8%)	1,639 (93.4%)	5,247 (93.2%)
Total	2,112	1,766	1,754	5,632
**Posttraumatic stress disorder**				
Negative	1,020 (48.3%)	838 (47.5%)	850 (48.5%)	2,708 (48.1%)
Positive	1,092 (51.7%)	928 (52.5%)	904 (51.5%)	2,924 (51.9%)
Total	2,112	1,766	1,754	5,632
**Major depression and posttraumatic stress disorder**				
Negative	1127 (53.4%)	933 (52.8%)	939 (53.5%)	2999 (53.2%)
Positive	985 (46.6%)	833 (47.2%)	815 (46.5%)	2633 (46.8%)
Total	2,112	1,766	1,754	5,632
Depression symptoms (BDI II) score [Mean ± SD *(n)*]	29 ± 10.4 (*n =* 2,112)	29 ± 10.4 (*n =* 1,766)	28.9 ± 10.3 (*n =* 1,754)	29 ± 10.4 (*n =* 5,632)
Depression symptoms [Median (min-max)]	28 (0.0–60)	28 (0.0–60)	28 (1–60)	28 (0.0–60)
PTSD symptoms (PCL-5) [Mean ± SD *(n)*)	43.5 ± 17.3 (*n =* 2,112)	43.2 ± 17.2 (*n =* 1,766)	43.5 ± 17.2 (*n =* 1,754)	43.4 ± 17.2 (*n =* 5,632)
PTSD symptoms [Median (min-max)]	42 (0.0–80)	42 (0.0–80)	42 (0.0–80)	42 (0.0–80)
PC-PTSD (Mean ± SD *(n)*)	2.21 ± 2.27 (*n =* 2,112)	2.22 ± 2.27 (*n =* 1,766)	2.16 ± 2.27 (*n =* 1,754)	2.2 ± 2.27 (*n =* 5,632)
PC-PTSD [Median (min-max)]	1 (0.0–5)	2 (0.0–5)	1 (0.0–5)	1 (0.0–5)
**HIV**				
Negative	1,283 (60.7%)	1,074 (60.8%)	1,044 (59.5%)	3,401 (60.4%)
Positive	829 (39.3%)	692 (39.2%)	710 (40.5%)	2,231 (39.6%)
Total	2,112	1,766	1,754	5,632
**Other Medical Co-morbidity** ^**^				
Negative	1,918 (90.8%)	1,605 (90.9%)	1,592 (90.8%)	5,115 (90.8%)
Positive	194 (9.2%)	161 (9.1%)	162 (9.2%)	517 (9.2%)
Total	2,112	1,766	1,754	5,632
**Number of different trauma categories** ^***^				
0	165 (7.8%)	140 (7.9%)	141 (8.0%)	446 (7.9%)
1	573 (27.1%)	468 (26.5%)	482 (27.5%)	1,523 (27.0%)
2	916 (43.4%)	780 (44.2%)	756 (43.1%)	2,452 (43.5%)
3+	458 (21.7%)	378 (21.4%)	375 (21.4%)	1,211 (21.5%)
Total	2,112	1,766	1,754	5,632
Total number of lifetime traumas [Mean ± SD *(n)*]	4.10 ± 2.98 (*n =* 2,112)	4.08 ± 2.92 (*n =* 1,766)	4.04 ± 2.98 (*n =* 1,754)	4.07 ± 2.96 (*n =* 5,632)
Total number of lifetime traumas [Median (min-max)]	4 (0.0–18)	4 (0.0–18)	4 (0.0–18)	4 (0.0–18)
Lifetime crime-related traumas [Mean ± SD *(n)*]	1.22 ± 1.14 (*n =* 2,112)	1.24 ± 1.14 (*n =* 1,766)	1.22 ± 1.14 (*n =* 1,754)	1.23 ± 1.14 (*n =* 5,632)
Lifetime crime-related traumas [Median (min-max)]	1 (0.0–4)	1 (0.0–4)	1 (0.0–4)	1 (0.0–4)
Lifetime disaster related traumas [Mean ± SD *(n)*]	2.41 ± 1.9 (*n =* 2,112)	2.39 ± 1.86 (*n =* 1,766)	2.36 ± 1.88 (*n =* 1,754)	2.39 ± 1.88 (*n =* 5,632)
Lifetime disaster related traumas [Median (min-max)]	2 (0.0–10)	2 (0.0–10)	2 (0.0–10)	2 (0.0–10)
Lifetime physical or sexual traumas [Mean ± SD *(n)*]	0.40 ± 0.82 (*n =* 2,112)	0.38 ± 0.80 (*n =* 1,766)	0.40 ± 0.83 (*n =* 1,754)	0.39 ± 0.82 (*n =* 5,632)
Lifetime physical or sexual traumas [Median (min-max)]	0.0 (0.0–6)	0.0 (0.0–5)	0.0 (0.0–6)	0.0 (0.0–6)
Lifetime other traumas [Mean ± SD *(n)*]	0.07 ± 0.25 (*n =* 2,112)	0.07 ± 0.25 (*n =* 1,766)	0.06 ± 0.25 (*n =* 1,754)	0.066 ± 0.25 (*n =* 5,632)
Lifetime other traumas [Median (min-max)]	0.0 (0.0–1)	0.0 (0.0–1)	0.0 (0.0–1)	0.0 (0.0–1)
**Number of participants in an intimate relationship**				
Not in intimate relationship	1,014 (48.0%)	857 (48.5%)	840 (47.9%)	2,711 (48.1%)
In an intimate relationship	1,098 (52.0%)	909 (51.5%)	914 (52.1%)	2921 (51.9%)
Total	2,112	1,766	1,754	5,632
**History of intimate partner violence among partners**				
No history of intimate partner violence	447 (40.7%)	384 (42.2%)	356 (38.9%)	1187 (40.6%)
History of intimate partner violence	651 (59.3%)	525 (57.8%)	558 (61.1%)	1734 (59.4%)
Total	1,098	909	914	2,921

^*^Datasets were created by randomly dividing observations and this table reports on individuals who may have contributed observations to more than one dataset; ^**^Hypertension, diabetes, tuberculosis, syphilis, hypothyroidism, hyperthyroidism; ^***^Trauma History Questionnaire.

### Sample population

SMART DAPPER enrollment eligibility required a positive diagnosis of major depression and/or PTSD, using corresponding MINI modules. The PCL-5 and PC-PTSD-5 were collected at baseline, 6 weeks and 3, 6, 9, 12, 18, 24, 30 months post-baseline. To evaluate a distribution with more negative diagnoses, the data set for this project included 13,099 records collected between September 2020 and March 2022.

### Analysis

We first randomly divided our dataset into a 30% test dataset and 70% development dataset. In our 70% development dataset we further randomly subdivided it into training (2/3) and validation (1/3) subsets. The validation subset was used to choose the optimal value of the shrinkage parameter in each of the regression models. After evaluating the performance of the models in the development dataset we chose a small number of models to balance performance and brevity of the screener. The test dataset was reserved to measure the performance of this small set of final models in an unbiased way.

We used the LASSO (least absolute shrinkage and selection operator) to select our models since it is a modern machine learning method that allows simultaneous variable selection and coefficient estimation. We preferred the LASSO over other machine learning methods (e.g., random forests) because of the ease of interpretation and ease of application in low resource settings.

We used PTSD as determined by the MINI PTSD as our gold standard outcome and considered predictor sets of (a) the 20 PCL questions, (b) the 5 PC-PTSD-5 questions (since this is an accepted short screen by itself) and (c) all 25 questions from both PCL and PC-PTSD. We used the individual questions as predictors to give maximum flexibility to the fitting and to allow consideration of screening tools with very few questions. A logistic regression model using LASSO was fit by minimizing the Average Squared Error (ASE) in the validation data.

We then examined the best fitting models and considered simplified versions either by rounding coefficients to integers to make them easier to use in practice or making them binary (above or below a cut-point). We evaluated their performance using validation data by calculating area under the receiver operating characteristic (AUROC) curve and sensitivities and specificities at various cutoffs.

Finally, we carried forward models that balanced ease of use and performance and assessed their performance using the reserved test dataset. The performance was assessed both overall and by subgroups defined by sex, age and trauma exposure. All analyses were conducted using SAS Version 9.4.

## Results

Overall, participants ranged in age from 18 to 85 years with an average age of 35.8 (11.0) and were predominantly female [*n* = 1,785 (91.1%)]. The training, validation and hold-out test datasets were very similar, Table 1.

### Lasso fitting

**Figure 1:**
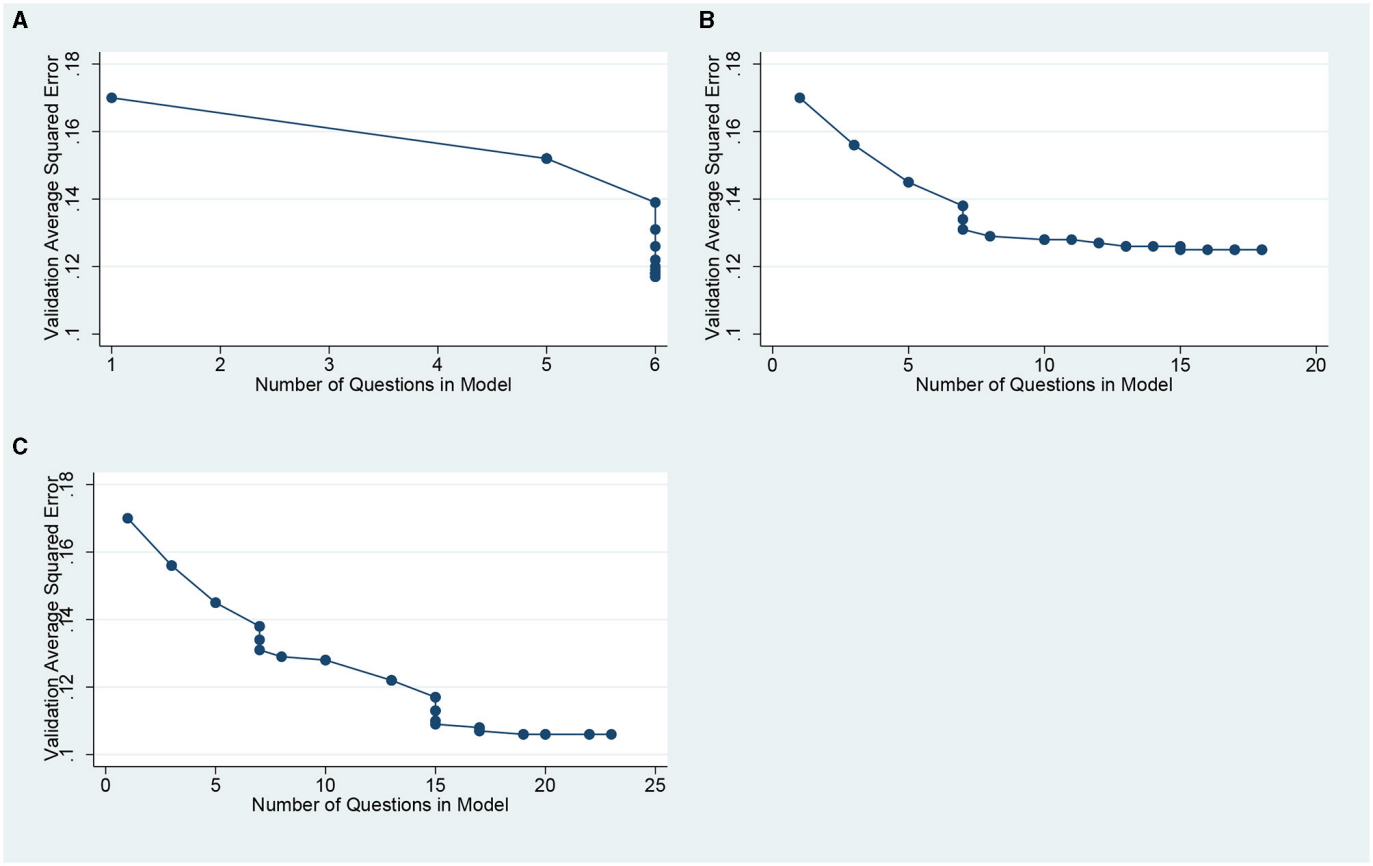
Averaged squared error vs. number of questions included in the LASSSo fit. **(A)** for the PC-PTSD questions only **(B)** for the PCL questions only. **(C)** for the combined PCL and PC-PTSD questions.

Figure 1 shows the Average Squared Error (ASE) as questions were added to the model for the PC-PTSD questions (1a), the PCL questions (1b) and the combined set of questions (1c). Each individual dot (Figure 1) is a separate LASSO model fit with different shrinkage parameters. The number of questions in the model is indicated on the horizontal axis. For the combined set of questions (1c), the optimal model contained 22 of the 25 questions, but the first nine questions entered in the model all came from the PCL. For the PCL questions only (1b), the optimal model contained 17 of the 20 questions and for the PC-PTSD questions, the optimal model contained all five questions. Table 2 gives the details of the three sequential LASSO fits.

**Table 2:** Coefficients for variables selected in the three LASSO model fits.

**Variable**	**Coefficient in PCL only model**	**Coefficient in PC-PTSD only model**	**Coefficient in combined model**	**Description**
PCL 1	0.21		0.19	Repeated, disturbing, and unwanted memories of the stressful experience
PCL 2	0.12		Not selected	Repeated, disturbing dreams of the stressful experience
PCL 3	0.096		0.11	Suddenly feeling or acting as if the stressful experience were actually happening again
PCL 4	Not selected		Not selected	Feeling very upset when something reminded you of the stressful experience
PCL 5	−0.019		−0.025	Having strong physical reactions when something reminded you of the stressful experience
PCL 6	0.067		0.049	Avoiding memories, thoughts, or feelings related to the stressful experience
PCL 7	0.15		0.13	Avoiding external reminders of the stressful experience
PCL 8	−0.093		−0.033	Trouble remembering important parts of the stressful experience
PCL 9	0.036		0.018	Having strong negative beliefs about yourself, other people, or the world
PCL 10	0.054		0.023	Blaming yourself or someone else for the stressful experience or what happened after it
PCL 11	0.19		0.10	Having strong negative feelings such as fear, horror, anger, guilt, or shame
PCL 12	0.10		0.12	Loss of interest in activities that you used to enjoy
PCL 13	0.11		0.076	Feeling distant or cut off from other people
PCL 14	0.088		0.062	Trouble experiencing positive feelings
PCL 15	−0.040		Not selected	Irritable behavior, angry outbursts, or acting aggressively
PCL 16	−0.27		−0.13	Taking too many risks or doing things that could cause you harm
PCL 17	Not selected		−0.0078	Being “super alert” or watchful or on guard
PCL 18	−0.042		−0.066	Feeling jumpy or easily startled
PCL 19	Not selected		0.0062	Having difficulty concentrating
PCL 20	0.28		0.25	Trouble falling or staying asleep
PC-PTSD 1		0.89	0.65	Have you had nightmares or thoughts about the event(s) when you did not want to?
PC-PTSD 2		0.38	0.52	Have you tried hard not to think about the event(s) or went out of your way to avoid situations that reminded you of the event(s)?
PC-PTSD 3		0.77	0.50	Have you been constantly on guard, watchful, or easily startled?
PC-PTSD 4		0.79	0.51	Have you felt numb or detached from people, activities, or your surroundings?
PC-PTSD 5		0.52	0.13	Have you felt guilty or unable to stop blaming yourself or others for the event(s) or any problems the event(s) may have caused?

Area under the ROC curve for selected models using the validation data (Table 3).

**Table 3:** Values of area under the receiving operating characteristic curve (AUROC) using validation data and hold-out test data.

**Question type/method of combining questions**	**Number of questions**	**AUROC validation data**	**AUROC hold-out test data**
PCL	2	0.84	0.85
Likert scale values multiplied by regression coefficient and summed	4	0.85	0.86
	6	0.86	
	12	0.86	
PC-PTSD	2	0.77	
Likert scale values multiplied by regression coefficients and summed	4	0.79	
	5	0.79	
PCL additive	2	0.83	0.85
Likert scale values summed	4	0.85	0.86
	6	0.86	
PCL rounded	2	0.84	0.85
Likert scale values mulitpled by rounded regression coefficient and summed	4	0.85	0.86
	6	0.86	
PCL 3 or above	2	0.71	
Likert scale values converted to binary scale (<3 = 0; ≥ 3 = 1) and summed	4	0.75	
	6	0.78	
PCL 2 or above	2	0.80	
Likert scale values converted to binary scale (<2 = 0; ≥ 2 = 1) and summed	4	0.82	
	6	0.84	

Since the analysis using the combined set of questions did not enter any of the PC-PTSD questions until the 10th question, it suggested we might prefer to base the screener on just the PCL question set. Also, since the curves in Figures 1A, C showed the fastest reduction in ASE with very few questions in the model, it suggests we might achieve good performance with very few questions. Accordingly, we calculated the AUROC for a number of models: (a) the best two-question, four-question, six-question and 12-question screener based on the PCL questions, and (b) the best two-question, four-question and the full set of PC-PTSD questions. The values of AUROC are given in Table 3 under the headings of PCL and PC-PTSD. As expected, the questions based on the PCL performed much better than the PC-PTSD. Even using the full five questions from PC-PTSD only achieved an AUROC of 0.79.

Table 3 also shows that there is very little performance lost by using a short screener. The model using only 2 questions had an AUROC of 0.84, only slightly less than the model using 12 questions (AUROC of 0.86). We therefore explored simplified versions of the PCL screeners, adding the values of the questions (“PCL additive” in Table 3) or by rounding the LASSO fit coefficients to round integers (“PCL rounded” in Table 3). In all cases, simply adding the values of the coefficients performed nearly as well as using the LASSO coefficients. We also explored counting how many of the questions were equal to or above 3 (“PCL 3 or above” in Table 3) or how many of the questions were equal to or above 2 (“PCL 3 or above” in Table 3). Those performed less well than adding the Likert scale values.

### Assessment of final models using the hold-out test dataset

The excellent performance of the simplified versions of the short screeners meant that we had very few final models to assess using the hold-out test data. Those were the two and four question versions using the PCL and the corresponding additive and rounded versions. The AUROCs for those models are given in Table 3. The simple screener, which adds the Likert scale values for two PCL-5 questions—Repeated, disturbing, and unwanted memories of the stressful experience (PCL-5 item #1) and Trouble falling or staying asleep (PCL-5 item #21)—had excellent performance, with an AUROC of 0.85, slightly better even than the training data.

### Assessment of final model by subgroup

Ideally, a screening tool will work well across different subgroups of a population. We therefore calculated the AUROCs using the hold-out test data separately for key subgroups. Men and women had AUROCs of 0.86 and 0.85, respectively. When broken down by age categories (18–85) the AUROCs were 0.84, 0.85, 0.86, and 0.84, respectively. When broken down by number of categories of trauma (0, 1, 2, 3 or more) the AUROCs were 0.78, 0.83, 0.86, and 0.86, respectively. Except for the no-trauma case, these were all comparable to the overall performance. While PTSD is highly co-morbid with depression (24, 25) evaluation of participants with only PTSD diagnosis (MDE negative) could provide useful information on the performance of the algorithms. PTSD instruments measure several symptoms of depression given some overlap of criteria. We therefore conducted a subgroup analysis comparing participants with PTSD and no MDE to all other combinations (PTSD and MDE, MDE alone and neither MDE nor PTSD). The performance of the screener was strong in both the PTSD only group (AUROC of 0.864) and in the remainder (AUROC of 0.849).

## Discussion

Penalized regression analysis suggested that a pragmatic and simple screening tool that adds the Likert scale values from two PCL-5 questions pertaining to intrusive thoughts of the stressful experience and insomnia worked well across subgroups defined by age, sex, and number of categories of trauma experienced. Intrusive thoughts and insomnia may be strong predictors of PTSD in this population.

Interestingly, these findings align with emerging data on risk factors associated with PTSD. An observational study of Emergency Department patients in Oxford, UK showed that sleep disruption immediately following trauma exposure was significantly associated with greater numbers of intrusive memories and higher risk of PTSD 2 months later (26). A recent meta-analytic review of eight experimental studies involving planned trauma exposure and sleep manipulation found that sleep reduced intrusive memory frequency (27). Researchers hypothesize that sleep disruption interferes with memory consolidation, which leads to more intrusive memories and higher risk of PTSD.

A priori we expected that the PC-PTSD would perform well, given its strong validation data, and wide-spread use, including LMIC settings. In the SMART-DAPPER Kenyan primary care population, the PC-PTSD did not correlate well with PTSD as diagnosed by the MINI.

### Implications

Within the past few years, the full PCL-5 has been validated in Rwanda, Africa (28). Given the strong discrimination metrics observed in this study with 2 items from the PCL-5 in Kenya, the utility of this brief PTSD screening tool may be regionally generalizable, and may also be useful in LMICs outside of SSA.

## Limitations

There are limitations to consider. Most notably, the algorithms were trained and tested using the SMART DAPPER study data and might show bias to the population enrolled in the study. For example, given the variability of PTSD symptom expression across cultures (16), the results may not generalizable outside of this study population. Further research on the proposed PTSD screener in other parts of Sub-Saharan Africa and international locations would provide valuable information on the generalizability to other contexts and populations. We also note that the SMART DAPPER study consisted primarily of females and may therefore lack generalizability to male populations. The study was open to males and females—aiming to match “real life” conditions of those seeking treatment in a primary care setting, we refrained from enriching the sample to achieve gender balance. The effect of gender on health seeking behavior is well established, with psychological, sociological and programming biases cited as potential sources drivers of low engagement of men (29–31). Future evaluations of this screener should include populations with higher male healthcare seeking behavior.

## Conclusion

A 2-item short version derived from the PCL-5 had excellent performance for identifying probable PTSD in our study population. This scale was significantly more accurate than a commonly used instrument for PTSD screening, the PC-PTSD. This tool has the potential to improve screening for PTSD in high-burden SSA clinical populations. Accurate, efficient screening would facilitate narrowing of the current PTSD treatment gap and improved population health.

## Data availability statement

The datasets presented in this study can be found in online repositories. The names of the repository/repositories and accession number(s) can be found at: National Institute of Mental Health Data Archive (NDA) (Collection ID: 3196).

## Ethics statement

The studies involving humans were approved by KNH-UON ERC, UCSF Human Research Protection Program Institutional Review Board (IRB), Pharmacy and Poisons Board (PPB), Kenya, and National Commission For Science, Technology and Innovation. The studies were conducted in accordance with the local legislation and institutional requirements. The participants provided their written informed consent to participate in this study.

## Author contributions

SM: Conceptualization, Formal analysis, Funding acquisition, Investigation, Project administration, Supervision, Writing—original draft, Writing—review & editing. MM: Funding acquisition, Investigation, Project administration, Supervision, Writing—review & editing. LO: Investigation, Supervision, Writing—review & editing. TN: Investigation, Writing—review & editing. DM: Investigation, Writing—review & editing. DOn: Investigation, Writing—review & editing. DA: Investigation, Writing—review & editing. GR: Supervision, Writing—review & editing. AO: Project administration, Supervision, Writing—review & editing. RO: Data curation, Supervision, Writing—review & editing. JW: Supervision, Writing—review & editing. EO: Supervision, Writing—review & editing. PM: Project administration, Writing—review & editing. DOl: Supervision, Writing—review & editing. RW: Supervision, Writing—review & editing. AM: Investigation, Writing—review & editing. JK: Investigation, Writing—review & editing. CC: Investigation, Writing—review & editing. DB: Investigation, Writing—review & editing. GA: Investigation, Writing—review & editing. RB: Project administration, Supervision, Writing—review & editing. CJ: Data curation, Formal analysis, Methodology, Validation, Writing—review & editing. CM: Data curation, Formal analysis, Investigation, Methodology, Validation, Writing—review & editing. SN: Investigation, Writing—review & editing.
